# Similarity of Fibroglandular Breast Tissue Content Measured from Magnetic Resonance and Mammographic Images and by a Mathematical Algorithm

**DOI:** 10.1155/2014/961679

**Published:** 2014-07-15

**Authors:** Fatima Nayeem, Hyunsu Ju, Donald G. Brunder, Manubai Nagamani, Karl E. Anderson, Tuenchit Khamapirad, Lee-Jane W. Lu

**Affiliations:** ^1^Division of Human Nutrition, Department of Preventive Medicine and Community Health, The University of Texas Medical Branch, 700 Harborside Drive, Galveston, TX 77555-1109, USA; ^2^Division of Biostatistics, Department of Preventive Medicine and Community Health, The University of Texas Medical Branch, Galveston, TX 77550-1147, USA; ^3^Department of Academic Computing, The University of Texas Medical Branch, Galveston, TX 77555-1035, USA; ^4^Department of Obstetrics and Gynecology, The University of Texas Medical Branch, Galveston, TX 77555, USA; ^5^Houston Bay Area Fertility Center, 9C Professional Park Drive, Webster, TX 77598, USA; ^6^Department of Radiology, The University of Texas Medical Branch, Galveston, TX 77555, USA; ^7^Breast Center, The Methodist Willowbrook Hospital, Houston, TX 77070, USA

## Abstract

Women with high breast density (BD) have a 4- to 6-fold greater risk for breast cancer than women with low BD. We found that BD can be easily computed from a mathematical algorithm using routine mammographic imaging data or by a curve-fitting algorithm using fat and nonfat suppression magnetic resonance imaging (MRI) data. These BD measures in a strictly defined group of premenopausal women providing both mammographic and breast MRI images were predicted as well by the same set of strong predictor variables as were measures from a published laborious histogram segmentation method and a full field digital mammographic unit in multivariate regression models. We also found that the number of completed pregnancies, C-reactive protein, aspartate aminotransferase, and progesterone were more strongly associated with amounts of glandular tissue than adipose tissue, while fat body mass, alanine aminotransferase, and insulin like growth factor-II appear to be more associated with the amount of breast adipose tissue. Our results show that methods of breast imaging and modalities for estimating the amount of glandular tissue have no effects on the strength of these predictors of BD. Thus, the more convenient mathematical algorithm and the safer MRI protocols may facilitate prospective measurements of BD.

## 1. Introduction

Breast density (BD) reflects the proportion of fibroglandular tissue in the breast and is one of the strongest independent predictors of breast cancer risk [1–4]. The most widely used method for measuring BD is the histogram segmentation method (HSM) using mammographic images, as pioneered by Byng et al. [5]. HSM is a user-guided graphic interactive thresholding method that is semiautomatic and computer-assisted but is also time-consuming, labor intensive, and subjective.

Mammography is designed to detect early breast cancer rather than to measure BD, and the radiation dose required for detecting cancer is greater for women with dense breasts. The multiple possible variations in instrument settings can confound the use of mammograms for BD estimates, and for this reason phantoms or step-wedge standards are included for calibration of mammography when measuring volumetric density [6, 7]. Individualized imaging parameters are routinely stored in the DICOM header of the mammogram report. We developed a mathematical model (MATH) that uses a substantial number of these individualized imaging parameters to automatically compute BD upon mammogram acquisition, thereby omitting the laborious HSM procedure [8, 9]. The full field digital mammography (FFDM) unit also routinely estimates and records percent glandular breast tissue. This estimate is used by the FFDM unit to optimize radiation dose for final screening mammography.

Mammography projects a 3-dimensional (3D) tissue into a 2-dimensional (2D) image. Thus, area measured from a 2D image can be expected to deviate from 3D volumes. Shepherd et al. [10] developed compressible breast phantoms with known and varying breast composition (e.g., 0–80% glandular tissue) which were imaged together with each mammogram. The density in the phantom was then used to calibrate the density in the pixels of a 2D mammogram. This algorithm considers the effect of breast compression on breast density. Using this approach, glandular volume measurements were found to be more strongly associated with breast cancer risk than with glandular area measurements alone [10]. We have shown that total volume (TV), glandular volume (GV), and adipose (fat) volume (FV) of the breast can be easily and reasonably approximated by multiplying the fat and gland tissue areas of the mammogram by the compression thickness of the breast as recorded in the mammogram DICOM header report [9].

The common use of mammography for breast cancer screening is due in part to its low cost. Limitations include a 2D projection of the compressed breast. Due to radiation exposure, mammography is not commonly applied to women less than 45 years old, unless medically indicated. Lack of mammographic imaging data in younger women makes it difficult to assess the role of BD in women of younger age in predicting later-in-life breast cancer risk. Thus, there is increased interest in the use of magnetic resonance imaging (MRI) for acquiring breast images, because it avoids radiation exposure and provides 3D images.

Several feasible MRI protocols for measuring fibroglandular tissue are available and the imaging protocols are typically a variation of clinically used T1 relaxation-rate MRI protocols, with or without fat suppression [9]. Four alternative conceptual approaches for estimating the volume of breast glandular tissue from MRI data have been investigated, namely, (I) segmentation of glandular and fatty tissues by an interactive thresholding algorithm [11, 12], (II) use of a clustering algorithm [13, 14], (III) a logistic function approach [15], or (IV) a curve-fitting algorithm [9].

We previously showed that breast glandularity measured as percent glandular tissue (%-G) (commonly referred to in the literature as percent breast density), glandular tissue volume (GV), fat volume (FV), and total volume (TV) from mammographic and MRI images were highly correlated with one another by ordinary least square regression (*R* ^2^) and intraclass correlation (ICC) analyses (all correlation coefficients > 0.75) [9]. Because there is no “gold standard” for measuring breast tissue composition, to further assess the usefulness of these measurement methods, we compared the similarities among patterns of biological predictors of BD measured by two breast images (MRI and mammography) and five breast density estimation methods.

## 2. Materials and Methods

### 2.1. Study Design

The main purpose of this study was to investigate the effects of methods of imaging the breast and measuring BD on biological features that may be associated with BD. BD measures by three new methods (MATH and two MRI methods) and by a FFDM unit were compared to that by a widely used HSM. The two MRI methods were a gradient-echo pulse sequence (3DGRE) and a fat suppressing, fast inversion spin echo pulse sequence (STIR). Data for dependent and independent study variables included only those that could be measured objectively. The study was compliant with HIPAA regulations and was approved by the Institutional Review Board of the University of Texas Medical Branch and the Human Research Protection Office of the US Army Medical Research and Materiel Command. Written informed consent was obtained from all subjects.

Healthy premenopausal women of all major races/ethnicities, living within 80 km of Galveston, Texas, were recruited, using webmail, posted advertisements, and postal mail. Women were 30 to 40 years old with regular monthly menstrual cycles. Subjects who were breast feeding, pregnant, expecting to become pregnant, or had used any type of contraceptive medication (oral, injection, or patch) within the prior 6 months were excluded. Multiple fasting blood samples from two separate menstrual cycles, one screening digital mammogram and two breast MR images, were all obtained during the same or separate luteal phase not more than 3 menstrual cycles apart. Only images of the left breast were analyzed in this study. Anthropometric and reproductive variables were also obtained.

### 2.2. Main Study Outcomes (Dependent Variables) and Their Measurement Methods

There were four BD outcomes of interest, %-G, GV, FV, and TV, for multivariate regression model analyses. These were obtained in a sample of 320 women by five methods, three from 2D mammography (HSM, MATH, and FFDM) and two from 3D MRI (3DGRE and STIR). The total breast is readily isolated from surrounding background and tissue on both mammographic and MR images. Mammography generated one image and one total breast area/volume for analysis by HSM, MATH, and FFDM, and MRI generated two images and two total breast volume estimates using 3DGRE and STIR.

### 2.3. Digital Mammography Methods (HSM, MATH, and FFDM)

We developed software in-house for BD analyses using digital mammograms [8] by applying the HSM algorithm of Byng et al. [5]. Briefly, the unprocessed (raw) and the processed digital mammograms were acquired using a GE Senographe 2000D FFDM unit (General Electric Healthcare Institute, Waukesha, WI). Craniocaudal (CC) and mediolateral-oblique (MLO) views of the left and right breasts were acquired. The raw CC view of the left breast was quantified for total breast area (*T*_AREA_), fibroglandular area (*G*_AREA_), fat (adipose) area (*F*_AREA_), and %-breast density (%-G) [8]. The processed images were not suitable for BD analyses, because the window and level settings varied between mammograms in order to provide sharp contrast between dense and nondense tissues to meet diagnostic needs for detecting breast cancer. However, the raw images allowed us to apply a consistent algorithm for setting the window and level for image viewing and dense tissue segmentation and were used for BD estimation.

Briefly, the breast tissue region of interest (ROI) was isolated from the chest wall and muscle to obtain the total breast area for each mammogram and for generating a signal-intensity histogram of the breast ROI. With the aid of graphical user-interactive software, an analyst subjectively selected suitable signal intensity from the histogram as a threshold that best segmented glandular area (*G*_AREA_) from fat tissue area (*F*_AREA_). For the HSM method, total breast area (*T*_AREA_) is the sum of *G*_AREA_ and *F*_AREA_ and %-G is calculated as the ratio of *G*_AREA_/*T*_AREA_. This analyst-dependent process took about 30 min.

GV, FV, and TV were the products of the respective tissue mammogram areas, the compression thickness, and a unit correction factor. For the viewing geometry of our imager, the unit correction factor for converting pixel area to mL (or cc) was 9.96, as described previously [9]. The DICOM header report included both preexposure and final exposure compression thickness. Preexposure compression thickness was used to estimate volumes, as follows:

(1) %-G=GAREAGAREA+FAREA=GAREATAREA,GV=9.96·GAREA·compression  thickness,FV=9.96·FAREA·compression  thickness,TV=GV+FV=9.96·TAREA·compression  thickness.

For the MATH method, %-G was computed using the following multivariate regression model equation that included image data from postmenopausal and other premenopausal women not involved in this study [8, 9]:

(2) %-G =481.33−0.0057·preexposure  dose  +1.2305·preexposure  thickness  −0.094·radiation  dose  +5.2056·pre-exposure  kvp  −0.0599·anatomical  mean  intensity  −0.0192·Thresh−2.0223·final  exposure  thickness  −0.049·compression  force  −37220·detector  sensitivity  −1.9863·filter  material+25.314·anode  material.

All variables in (2) are used by the digital mammography unit to produce a screening image and are strong and significant predictors of BD. The DICOM tag for each variable for the specific mammographic unit used for this study has been described previously [8]. (Note: the DICOM tags may differ for different scanners.) The data for each imaging variable was retrieved from the mammogram DICOM header. The filter material and anode material were either molybdenum or rhodium, which were coded as 1 or 0, respectively, for calculating %-G. The %-G obtained from (2) was then used to calculate GV and FV for the MATH method using the following approaches:

(3) $$\begin{array}{c} \text{GV}=\text{TV}\cdot \%\text{-G},\\ \text{FV}=\text{TV}\cdot \left(1-\%\text{-G}\right). \end{array}$$

The FFDM unit itself gives an estimate of percent breast density for each mammogram, which is also available from the mammogram DICOM header as “Raddose” and “precompo.” Values for Raddose are almost the same as for precompo. Raddose values were used to represent %-G from the FFDM unit for calculating GV and FV, according to (3).

### 2.4. Magnetic Resonance Imaging (MRI) Methods (3DGRE and STIR)

The 3DGRE and STIR breast MRIs were performed as described previously [9]. Briefly, subjects were scanned in a prone position using a 1.5-Tesla MR scanner (General Electric, Waukesha, WI). The 3DGRE, a gradient-echo pulse sequence, took 3 minutes to be completed, and the imaging parameters were repetition time/echo time (TR/TE) = 5.9/1.4 ms, flip angle = 10°, acquisition matrix size = 256 × 256, reconstruction matrix size = 512 × 512, number of excitation (NEX) = 2, field of view (FOV) = 28–35 cm, and slice thickness = 1.5 mm (interpolated). The STIR protocol, a fat suppressing, fast inversion spin echo pulse sequence, took about 15 minutes to be completed, and the imaging parameters were TR/TE = 6050/12.9 ms, flip angle = 90°, an inversion time of 150 ms, acquisition matrix = 256 × 192, reconstruction matrix = 256 × 256, FOV = 28–35 cm, and slice thickness = 2 mm with 0 gap. The image acquisition was interleaved and repeated three times. After a MRI procedure, a 3D volume-rendered breast model was generated for the left breast ROI from either the 3DGRE or STIR protocol, respectively [9].

### 2.5. Curve-Fitting and Estimation of Glandular Tissue from Breast MR Images

Details for the analysis of breast tissue volume in mL or cm^3^ have been described [9]. Briefly the final segmented 3D volume-rendered breast model was used to generate a histogram of MRI voxel signal intensity. The histogram was then used for Gaussian curve-fitting analysis using a commercially available peak-fitting program, PeakFit 4.0 (SyStat Software Inc., San Jose, CA). The curve-analysis estimated the relative distribution of areas under the adipose and glandular breast tissue curves of the histogram, respectively, based on the assumption that breast tissue contained only two compartments, that is, adipose and fibroglandular tissues.

The final segmented 3D volume-rendered breast model was also subjected to volume analysis for the resampled/reconstructed 3D model using GE 3D Advantage Windows Workstation software version 4.1 (GE Healthcare Institute, Waukesha, WI), as follows. The reconstructed voxel size is the size of voxel in mm in both *x*- and *y*-directions. The voxel ratio is the ratio between the size of the voxels in the *z*-direction and in the *x*-direction. The voxel size and the voxel ratio of the reconstructed 3D model were recorded in the model DICOM header report and were retrieved for calculating voxel volume (mm^3^) which is the product of voxel ratio and (reconstructed voxel size)^3^. This approach provided volume in mL (cm^3^) for each breast tissue for direct comparison with volume estimated from mammograms as described above.

### 2.6. Anthropometrics, Body Composition, and Reproductive Factors

Body weight (kg), height (m), body mass index (BMI = kg/m^2^), waist circumference (in cm at the umbilicus), and hip circumference (in cm at the widest point around the buttocks) were obtained. Additionally, total body mass, lean body mass, and fat body mass were measured in duplicate (before and after repositioning), with the subject in a supine position, using dual energy X-ray absorptiometry (DEXA) (Model Discovery A, Model QDR4500A, Hologic, Waltham, MA). Average values of duplicate measurements were used for statistical analyses. Demographic and reproductive information (race, ethnicity, ages of menarche, first pregnancy, last pregnancy, and the number of completed pregnancies) were obtained using a self-administered questionnaire.

### 2.7. Analyses of Hormones and Blood Chemistries

Multiple fasting venous blood samples, drawn between 8:00 and 10:00 a.m., and between 20 and 24 days after menstrual spotting, were analyzed for 17*β*-estradiol, progesterone, insulin, insulin-like growth factor-I (IGF-I), insulin-like growth factor-II (IGF-II), sex hormone binding globulin (SHBG), and C-reactive protein (CRP). Enzyme-linked immunosorbent assay (ELISA) kits were used for measuring serum CRP (sensitivity 1.6 ng/mL) and SHBG (sensitivity 0.61 nmol/L). Immunoradiometric assays (IRMA) were used to measure serum IGF-I (sensitivity 10 ng/mL) and serum IGF-II (sensitivity 12 ng/mL). Radioactive immunoassay (RIA) kits were used to measure plasma 17*β*-estradiol (sensitivity 7 pg/mL), plasma progesterone (sensitivity 0.1 ng/mL), and serum insulin concentrations (sensitivity 1.3 *μ*IU/mL). All immunoassays were performed using commercially available kits (Diagnostic System Laboratories, Inc., Webster, TX). The intra- and interassay coefficients of variation for all analytes were <10%. Means of serum hormone concentrations from different study visits were used for statistical analyses.

Numerous fasting serum analytes, including glucose, total cholesterol, high-density lipoprotein cholesterol (HDL), triglycerides, alanine aminotransferase (ALT), aspartate aminotransferase (AST), and alkaline phosphatase (ALP), were measured by a certified hospital clinical laboratory using VITROS 5.1 FS (Ortho-Clinical Diagnostics, Rochester, NY).

### 2.8. Statistical Analyses

Data are presented as means and 95% confidence intervals (95% CI) of the mean for continuous variables and as frequencies for the categorical variables (ethnicity and parity). Main outcomes-of-interest are presented as box plots (SigmaPlot 12, Systat Software, Inc., San Jose, CA).

In a sample of 137 subjects from whom blood chemistries and hormone data were available at the time of statistical analyses, univariate associations between dependent variables (%-G, GV, FV, and TV) and predictor variables were computed. Exploratory multivariate analyses between the dependent variables and predictor variables were performed by the GLMSELECT procedure in SAS (with stepwise, forward LAR and LASSO options) to select the best models with information criterion such as AIC, BIC, and Cp options. Good models will have small values of this criterion to select candidate predictors. GLMSELECT models were run with %-G, GV, FV, and TV as dependent variables together with a block of anthropometric measures (body weight, height, BMI, waist and hip circumference, and fat and lean body mass) or a block of blood chemistry variables (a lipid panel of cholesterol, HDL, LDL, VLDL, and triglycerides, liver enzymes of ALP, ALT, and AST, and hormones). Predictor variables, selected consistently in GLMSELECT models for all outcome variables of interest, were included in the final models. We are not aware of any prior studies examining the relationship between routinely measured blood chemistries and BD. Such relationships were explored in this study in a preliminary fashion because the liver metabolizes ovarian steroids, whole body adiposity affects liver function and breast cancer risk, and predictors of GV are few (for more details, see Section 4).

All models were adjusted for age and reproductive variables known to influence BD, such as age of menarche and number of completed pregnancies. IGF-I, IGF-II, 17*β*-estradiol, progesterone, SHBG, CRP, and insulin have been studied for association with BD and breast cancer risk, and they were included as predictor variables in the final multivariate models. There was no multicollinearity problem among variables in the final models as indicated by variance inflation factors (all <5).

The final multivariate model also included methods of measurement of BD as predictor variables and interaction terms between measurement methods and respective predictor variables. We performed similarity test procedures of *β*-estimates across methods of measurement by a deviance test or log likelihood test for comparing the full versus the nested models. Post hoc pairwise comparisons with false discovery rate (FDR) adjustment were used to assess differences [16]. The effects of measurement methods on multivariate regression models were validated in another sample of 320 women from whom demographic, anthropometric, and reproductive variables were available but not blood chemistries or hormones. A significance level of *α* = 0.05 was used in our analyses. The statistical analyses were performed using the SAS statistical software package version 9.2 (SAS Institute, Cary, NC). The scatter plot matrix that included histograms was generated using R software (http://cran.r-project.org/, version 3.1.0).

## 3. Results

**Figure 1:**
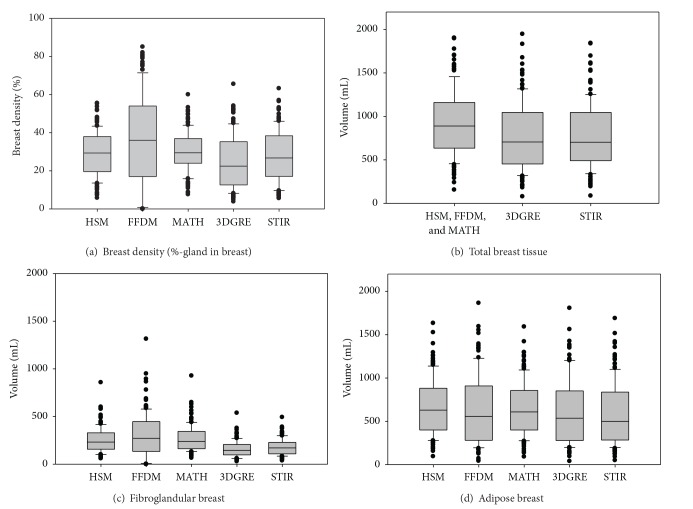
Interquartile box plots of breast density (a), total breast tissue volume (b), fibroglandular breast tissue volume (c), and adipose breast tissue (d) in 137 premenopausal women as measured by a histogram segmentation method (HSM), a full field digital mammography unit (FFDM) unit, a mathematical algorithm (MATH), a 3D gradient-echo (3DGRE) pulse sequence MRI, and a short tau inversion recovery pulse sequence (STIR) MRI. For the spread and distribution, consult histograms in Figure 2.

**Figure 2:**
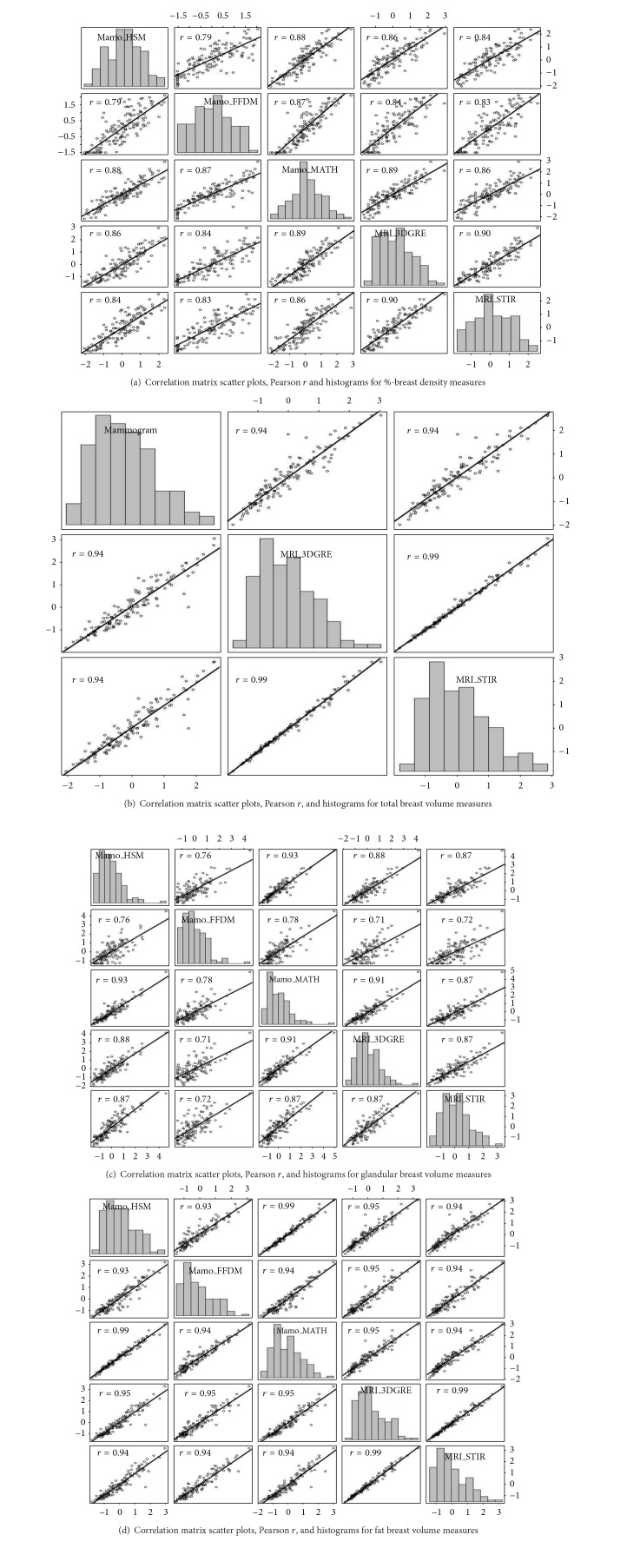
(a)–(d) Scatter plot matrix including Pearson *r* and regression line for pairwise correlation analyses between %-breast density (a), fibroglandular tissue volumes (b), adipose tissue volumes (c), and total breast tissue volumes (d) measured by five different methods. Diagonal boxes show histograms for each variable. Mamo_HSM (1st row and 1st column), histogram segmentation method using mammograms; Mamo_FFDM (2nd row and 2nd column), mammograms from full field digital mammography; Mamo_MATH (3rd row and 3rd column), mathematical algorithm for computing breast tissue content using mammograms; MRI_3DGRE (4th row and 4th column), 3-dimensional gradient-echo pulse sequence using MRI images; MRI_STIR (5th row and 5th column), short tau inversion recovery pulse sequence using MRI images. Units of measures for *x*-axis and *y*-axis are *Z* score (mean = 0, standard = 1) for (a)–(d). All data used for each pairwise correlation analysis are included within the graph ruler space. The bin width within each histogram is equally distributed within the column *x*-axis scale and frequency in *y*-axis is not labeled but represents relative distribution. For mean values, consult Figure 1.

The racial/ethnic composition of the study population was 54% non-Hispanic White, 30% Hispanic, and 16% African American.  Table 1 shows additional relevant characteristics of the subjects that were included in the study. Figures 1(a)–1(d) show the mean and interquartile box plots of %-G, TV (in mL), GV (in mL), and FV (in mL) measured by five different methods, HSM, the FFDM unit, MATH, 3DGRE, and STIR, as applicable. Figures 2(a)–2(d) show scatter plot matrices, including histograms (diagonal boxes), for four different BD measures, %-G, TV, GV, and FV, respectively. As shown, Pearson's correlation coefficients are high, ranging from 0.76 to 0.99 for pairwise correlation analyses in BD measured by the five methods [9]. Note that the distribution of %-G and GV from the FFDM unit tended to be wider; see box plots in Figures 1(a) and 1(c) and 2nd diagonal box histograms of Figures 2(a) and 2(c)).

The 2D mammography provides breast an area measure. Because fatty breast is more easily compressed than dense breast, this differential compression may bias %-breast density when estimated from mammograms. We correlated the area breast measure from mammograms with the volume measures from 3D MR images. Correlation coefficients of measures using areas with corresponding MRI volumes (from 3DGRE and STIR) were 0.83 for glandular area (*G*_AREA_), 0.88 for glandular mammographic volume (GV = *G*_AREA_ × compression thickness), ~0.93 for fatty breast area (*F*_AREA_), ~0.95 for fatty mammographic breast volume (FV = *F*_AREA_ × compression thickness), ~0.92 for total mammographic breast area (*T*_AREA_), and ~0.94 for total mammographic breast volume (TV = *T*_AREA_ ×  compression thickness) (results not graphed). Thus, conversion of mammographic area (pixel in mm^2^) to mammographic volume (cm^3^ or mL) resulted in slight improvement of correlation with MRI volumes. Note that the conversion of pixel from mammogram and voxel from breast MRI have all been corrected for viewing geometry of imagers to give mL.

### 3.1. Effects of Measurement Methods on Quantitative Breast Tissue Composition

Table 2 shows that mean %-G, TV, GV, and FV values did not differ when compared within the same breast imaging modality, but GV and TV did differ when compared between MRI and mammography measures. Interestingly, mean FV differed significantly only between STIR and HSM or MATH. For %-G, means of 3DGRE differed significantly from each of the three mammographic methods, while mean %-G of STIR did not differ from mean %-G of HSM or MATH but differed from %-G of the FFDM unit. In other words, %-G of the FFDM unit was different from all other %-G measurements.

There is no gold standard for calibrating BD, and the physics behind mammography and MRI differs. Therefore, it is important to know whether correlations with biological factors known to predict breast %-G, GV, FV, and TV are affected by measurement methods.

### 3.2. Pearson's Correlation Analyses Between BD and Biological Features

The univariate analysis results between dependent and independent variables are shown in Table 3. Pearson's correlation coefficients ranged from >0.2 to 0.8 (*P* < 0.0001 to 0.01) between %-G, FV, and TV, as measured by five different methods with all anthropometric variables except height, and with HDL, ALP, SHBG, and CRP. A consistent and significant linear correlation was observed only between CRP and GV (measured by HSM, MATH, 3DGRE, and STIR).

### 3.3. Effects of Measurement Methods on Regression Models of Breast Tissue Composition

The primary objective of our study was to investigate the effects of the five BD measurement methods (HSM, FFDM, MATH, 3DGRE, and STIR) on profiles of biological predictors of %-G, GV, FV, and TV.

Exploratory models were run to select strong predictors for inclusion in final multivariate models. Fat body mass, BMI, and waist-to-hip ratio were most frequently selected as predictor anthropometric variables by PROC GLMSELECT in the exploratory models. Due to strong collinearity, BMI, fat body mass, and waist-to-hip ratio were tested one at a time in the multivariate models. BMI was included in the final models, but it can be replaced by fat body mass with minimal change in the profiles and strength of significant independent predictors, that is, in terms of *β*-estimates, *P* values, and model *R* ^2^. In the sample of 137 subjects from whom blood chemistries were available at the time of statistical modeling, HDL, total cholesterol, ALP, ALT, and AST were most frequently represented by PROC GLMSELECT as significant predictor variables from blood chemistries. However, total cholesterol, HDL, and insulin were not significant independent predictors in multivariate models that included BMI or fat body mass and therefore were removed from the final regression models.

Predictor variables for BD, included in the final multivariate models, were BMI, age, age of menarche, and number of completed pregnancies (*n* = 320). Additionally, in a subset of 137 subjects, hormones (ALP, ALT, AST, SHBG, CRP, IGF-I, IGF-II, 17*β*-estradiol, and progesterone) and blood chemistries were included.  Table 4 shows, within multivariate models on the subset of 137 subjects, standardized *β*-estimates and standard errors (SE) of the estimates for %-G, GV, FV, and TV, respectively, using HSM as a reference for the measurement method, while Table 5 shows the results for the sample of 320 women from whom levels of blood chemistries and hormones were not yet analyzed.

### 3.4. Effects of Methods of Measurement on Predictors of Breast Composition

Within each multivariate analysis, an interaction term for each predictor variable with measurement methods was also included. All of the interaction terms between measurement methods and biological predictor variables by deviance or likelihood ratio tests were not significant (e.g., all *P* values were between 0.20 and 1.00), so the interaction terms were removed from the final multivariate regression models.  Table 4 shows the multivariate regression models using HSM as reference for the measurement method.

The first nested model within the multivariate model for %-G (Table 4) showed a significant association between %-G and BMI (*P* < 0.0001), number of completed pregnancies (*P* = 0.02), ALT (*P* = 0.02), AST (*P* = 0.001), progesterone (*P* = 0.04), and African-American race (*P* < 0.05). These associations were independent of BD measurement methods. The aggregate model *R* ^2^ for %-G was 0.54. The second regression model in Table 4 shows that fibroglandular tissue volume (GV) was significantly associated with number of completed pregnancies (*P* = 0.0004), AST (*P* = 0.05), CRP (*P* = 0.04), and progesterone (*P* = 0.02). Again, these associations were not affected by BD measurement methods. The aggregate model *R* ^2^ for GV was 0.29. The third model in Table 4 shows that the adipose breast tissue (FV) had a significant association with BMI (*P* < 0.0001), ALP (*P* = 0.04), and IGF-II (*P* = 0.004) that was also not affected by BD measurement methods. The aggregate model *R* ^2^ for FV was 0.71. The last model in Table 4 shows that the total breast volume (TV), as measured by digital mammography and two MRI protocols, was significantly associated with BMI (*P* < 0.0001), number of completed pregnancies (*P* = 0.01), and IGF-II (*P* = 0.02). This association was also not affected by BD measurement methods. The aggregate model *R* ^2^ for TV was 0.65. The strong and significant association between BD and anthropometric and reproductive variables found in the sample of 137 subjects was confirmed in the larger sample of 320 women (Table 5) from whom blood analytes were not available at the time of regression model analyses.

## 4. Discussion

We recently demonstrated that two mammography (HSM and MATH) and two MRI-based modalities (3DGRE and STIR) could reliably measure breast tissue composition (i.e., %-G, GV, FV, and TV), in that all intraclass correlation and regression coefficient values were >0.75 [9]. Because there is no gold standard for* in vivo* measurement of breast tissue content, and there are quantitative differences in estimates (Table 2) that may be due possibly to differences in radiologic imaging techniques, 2D and 3D image acquisition, or tissue segmentation methods, it is important to determine if the measurement methods have any influence on correlations with known determinants of BD. In this study, we show that biological predictors of BD in a sample of 30- to 40-year-old premenopausal women were strikingly similar across all five BD measurement methods, between two different radiologic imaging modalities, and were similar to those reported in older women [17–20]. Our results (Tables 4 and 5) suggest inference validity. Because the MATH method can compute BD automatically upon mammogram acquisition, it should be tested and validated further in future BD and breast cancer risk prediction studies in light of increasing use of digital mammography for breast cancer screening.

The strong predictors of %-G, FV, and TV found in our sample of younger women (30 to 40 years old) were, in general, in line with those reported in older women. Briefly, whole body adiposity is predictive for breast tissue adiposity, and it explained the major portion of the variances found in %-G, FV, and TV [17–20]. In contrast to adiposity being a dominant predictor of %-G, FV, and TV, few strong predictors of fibroglandular tissue (GV) volume were reported. Neither BMI nor other anthropometric variables were associated with GV in our study of premenopausal women or in older women from other studies [19–21].

Parity has been consistently reported to be negatively associated with GV [22–24], which was confirmed in this study by both mammography and MRI images. The strength of the negative association between parity and glandular tissue is not surprising and has been attributed to the glandular tissue remodeling known to occur after each pregnancy and lactation [25]. However, the negative association between parity and TV or FV of the breast (Table 4) is unexpected in multivariate models controlling for BMI (Tables 4 and 5) or total body fat (results not shown). Thus, the decrease in fibroglandular tissue volume in breast after each pregnancy and lactation was not accompanied by a corresponding increase in breast fat/adipose tissue volume, as has often been speculated in the literature [26]. This interesting finding requires further confirmation by other investigators. However, parity explained only a small percentage of the variance in GV. We also explored predictors of GV in routinely measured blood chemistries and hormones.

SHBG and CRP correlated strongly with %-G, TV, GV, and FV in correlation analyses (Table 3) but were not independent predictors of %-G, TV, or FV in multivariate models when adjusted for fat body mass or BMI. This is consistent with reports showing that SHBG predicts %-G and GV_, _but not after adjustment for BMI [27, 28]. This can be explained by our previous finding that anthropometric variables are independent predictors of SHBG and CRP [29, 30]. Circulating CRP, however, remained a strong and positive independent predictor for GV across all five methods of measurement after adjusting for fat body mass and BMI. Mammary gland involution and remodeling involve components of wound healing [31, 32]. CRP, being a marker of inflammation, may play a role in remodeling as its presence has been reported in nipple aspirate fluid [33]. However, CRP has not been associated with breast cancer risk in epidemiologic studies [34–36] even though inflammation also plays an important role in breast cancer risk [37].

Obesity and the metabolic syndrome have been implicated in breast cancer risk [38]. Liver enzymes, such as ALP, AST, and ALT, are clinically useful markers for the metabolic syndrome and other obesity-related conditions [39, 40]. These enzymes were predictors of breast composition in our exploratory GLMSELECT models, but not in the final models including BMI for FV and TV. AST remained an independent predictor for both GV and %-G. The mechanisms underlying the direct association of AST with GV and %-G need further studies. The association between progesterone, estradiol, IGF-I, and IGF-II with BD was also not affected by BD measurement methods. This lack of association was consistent with some but not all literature reports [27, 28, 41].

CRP, AST, progesterone, and the number of completed pregnancies are more strongly associated with amounts of glandular tissue than with breast adipose tissue. Fat body mass, ALT, and IGF-II appear to be more associated with the amount of breast adipose rather than with glandular tissue. Associations between CRP, ALT, and AST and breast tissue composition have not been reported previously, to our knowledge, and further studies will be necessary to illuminate the mechanisms involved.

The strengths of this study were the inclusion of a population of multiethnic, premenopausal subjects with strictly defined characteristics who were not using exogenous hormones. All study samples were obtained during luteal phases within a short interval. Mean levels of hormones and blood chemistries from multiple blood samples were used for statistical analyses. To our knowledge, no other studies have validated biological features predicting BD as measured by both mammography and MRI in the same study subjects. Weaknesses of the study include a relatively small number of subjects with available measures of blood chemistries and hormones, a narrow age range for inclusion, and the exclusion of postmenopausal women and breast cancer patients, thereby limiting inferences. The parameter fit coefficients for the MATH equation, while applicable for postmenopausal women as previously described [8, 9], may require calibration for different brands and models of full field digital mammographic units.

In summary, we found similarities among determinants of breast %-G, GV, FV, and TV measured by five different methods. Our results suggest that the two MRI protocols and the mathematical algorithm that we developed should be further tested in studies of risk factors related to BD and breast cancer. Importantly, the MATH method was able to adjust for the inherent manipulation of imaging parameters by the mammography unit. Whether MATH algorithm improves risk prediction studies of breast density or breast cancer risk deserves further study as it can automatically compute BD upon mammogram acquisition. The two MRI protocols are complimentary in image acquisition for adipose and gland tissue. The sensitivity and specificity of these methods in measuring the effects of interventions that may reduce breast density and breast cancer risk require further study.

## Figures and Tables

**Table 1:** General characteristics of the study subjects (*n* = 137).

	n (%, column)
Race/ethnicity	
White	74 (54%)
Hispanic	41 (30%)
Black	22 (16%)

	Mean (95% CI)

Demographics and anthropometrics	
Age, y	35.9 (35.4, 36.4)
Weight, kg	74.8 (72.3, 77.4)
Height, cm	161.6 (160.4, 162.7)
BMI, kg/m^2^	28.7 (27.8, 29.7)
Fat body mass, kg	28.2 (26.5, 29.9)
Lean body mass, kg	46.9 (45.8, 48.0)
Waist circumference, cm	87.3 (85.3, 89.4)
Hip circumference, cm	109.7 (107.7, 111.8)
Reproductive history	
Age at menarche, y	12.5 (12.2, 12.8)
Age at first birth, y	23.3 (22.5, 24.2)
Years since last pregnancy	7.3 (6.4, 8.1)
Pregnancy, completed	
Zero	18 (13.1%)
One	17 (12.4%)
Two	44 (32.1%)
Three and more	58 (42.3%)
Blood chemistry and hormones	
Triglycerides, mg/dL	110.2 (97.4, 123)
Cholesterol, mg/dL	178.6 (173.7, 183.6)
HDL, mg/dL	53.1 (51, 55.2)
Alkaline phosphatase (ALP), U/L	70.6 (67.6, 73.7)
Alanine aminotransferase (ALT), U/L	26.9 (25.2, 28.6)
Aspartate aminotransferase (AST), U/L	21.1 (19.9, 22.3)
Sex hormone binding globulin (SHBG), nmol/L	101.9 (94.9, 108.9)
C-reactive protein (CRP), mg/L	6.8 (5.5, 8.1)
Insulin, *µ*IU/mL	12.6 (11, 14.2)
Insulin-like growth factor I (IGF-I), ng/mL	291.6 (272.4, 310.7)
Insulin-like growth factor II (IGF-II), ng/mL	865.1 (824.7, 905.5)
17*β*-Estradiol, pg/mL	132.2 (125.6, 138.9)
Progesterone, ng/mL	10.1 (9.2, 10.9)

**Table 2:** Mean differences and 95% confidence interval in percent glandular tissue (%-G), gland volume (GV), fat volume (FV), and total breast volume (TV) by Tukey's test.

Methods compared	%-G	GV (mL)	FV (mL)	TV (mL)
MATH versus HSM^a^	1.1 (−2.85, 4.94)^b^	11.4 (−32.52, 55.26)	10.7 (−55.57, 77.06)	0 (−75.98, 75.98)
STIR versus 3DGRE	2.9 (−1.00, 6.81)	22.3 (−21.65, 66.29)	24.3 (−42.17, 90.71)	1.9 (−74.32, 78.21)
3DGRE versus HSM	4.5 (0.56, 8.37)∗	94.1 (50.09, 138.04)∗	51.9 (−14.54, 118.34)	146.0 (69.7, 222.23)∗
3DGRE versus MATH	5.5 (1.60, 9.42)∗	105.4 (61.38, 149.49)∗	41.2 (−25.40, 107.71)	146.0 (69.7, 222.23)∗
3DGRE versus FFDM	11.5 (7.57, 15.38)∗	138.0 (94.05, 181.99)∗	7.9 (−58.49, 74.38)	146.0 (69.7, 222.23)∗
STIR versus HSM	1.6 (−2.22, 5.34)	71.8 (27.94, 115.56)∗	76.2 (9.97, 142.36)∗	147.9 (71.93, 223.90)∗
STIR versus MATH	2.6 (−1.29, 6.50)	83.1 (39.23, 127.01)∗	65.4 (−0.89, 131.74)∗	147.9 (71.93, 223.90)∗
STIR versus FFDM	8.6 (4.68, 12.46)∗	115.7 (71.89, 159.51)∗	33.2 (−98.40, 33.98)	147.9 (71.93, 223.90)∗
FFDM versus HSM	7.0 (3.12, 10.90)∗	44.0 (0.14, 87.76)∗	44 (−22.24, 110.15)	0 (−75.98, 75.98)
FFDM versus MATH	6.0 (2.07, 9.86)∗	32.6 (−11.31, 76.47)	33.2 (−33.20, 99.53)	0 (−75.98, 75.98)

^a^HSM, histogram segmentation method; FFDM, full field digital mammography unit; MATH, mathematical algorithm; 3DGRE, 3D gradient echo; STIR, short tau inversion recovery.

^b^Mean (95% confidence interval).

∗Difference between means, significance at *P* ≤ 0.05 with false discovery rate.

**Table 3:** Pearson's correlation coefficients between dependent and selected independent variables of study population (*n* = 137).

Variables	Pearson's correlation coefficients																				
	Prob > |*r*| under H0: Rho = 0																				
	%-G					Total breast tissue				Glandular tissue						Fatty breast tissue					
	HSM	FFDM	MATH	3DGRE	STIR	HSM_Area_	HSM	3DGRE	STIR	HSM_Area_	HSM	FFDM	MATH	3DGRE	STIR	HSM_Area_	HSM	FFDM	MATH	3DGRE	STIR
Age	−0.06 0.49	−0.10 0.23	−0.09 0.30	−0.11 0.19	−0.07 0.44	−0.13 0.14	−0.12 0.16	−0.06 0.47	−0.08 0.35	−0.14 0.10	−0.13 0.14	−0.13 0.14	−0.14 0.10	−0.16 0.07	−0.13 0.14	−0.08 0.33	−0.09 0.30	−0.04 0.61	−0.09 0.30	−0.03 0.73	−0.06 0.52
Age at menarche	0.12 0.16	0.09 0.30	0.11 0.21	0.08 0.37	0.11 0.21	−0.10 0.26	−0.07 0.43	−0.07 0.39	−0.07 0.39	0.07 0.39	0.05 0.53	0.00 0.96	−0.01 0.91	0.01 0.88	0.05 0.55	−0.13 0.13	−0.10 0.24	−0.06 0.47	−0.08 0.36	−0.08 0.36	−0.09 0.29

Weight	−0.59 <0.0001	−0.57 <0.0001	−0.55 <0.0001	−0.62 <0.0001	−0.61 <0.0001	0.64 <0.0001	0.65 <0.0001	0.75 <0.0001	0.74 <0.0001	−0.08 0.35	0.04 0.63	−0.20 0.02	0.16 0.07	−0.02 0.80	0.09 0.31	0.72 <0.0001	0.75 <0.0001	0.74 <0.0001	0.73 <0.0001	0.78 <0.0001	0.76 <0.0001
Height	0.06 0.52	0.10 0.23	0.04 0.61	0.11 0.21	0.13 0.13	−0.16 0.06	−0.14 0.11	−0.13 0.13	−0.12 0.15	−0.09 0.30	−0.10 0.26	−0.03 0.74	−0.12 0.17	−0.04 0.67	−0.04 0.64	−0.14 0.09	−0.12 0.16	−0.12 0.18	−0.12 0.15	−0.13 0.14	−0.12 0.16
BMI	−0.61 <0.0001	−0.61 <0.0001	−0.57 <0.0001	−0.66 <0.0001	−0.66 <0.0001	0.70 <0.0001	0.70 <0.0001	0.80 <0.0001	0.78 <0.0001	−0.04 0.61	0.08 0.35	−0.18 0.03	0.20 0.02	0.00 0.96	0.11 0.22	0.77 <0.0001	0.79 <0.0001	0.78 <0.0001	0.78 <0.0001	0.83 <0.0001	0.80 <0.0001
Fat body mass	−0.61 <0.0001	−0.62 <0.0001	−0.60 <0.0001	−0.66 <0.0001	−0.67 <0.0001	0.69 <0.0001	0.72 <0.0001	0.82 <0.0001	0.81 <0.0001	−0.04 0.64	0.11 0.21	−0.21 0.02	0.23 0.01	0.03 0.75	0.12 0.18	0.76 <0.0001	0.80 <0.0001	0.80 <0.0001	0.79 <0.0001	0.85 <0.0001	0.83 <0.0001
Lean body mass	−0.44 <0.0001	−0.36 <0.0001	−0.35 <0.0001	−0.41 <0.0001	−0.38 <0.0001	0.42 <0.0001	0.44 <0.0001	0.48 <0.0001	0.47 <0.0001	−0.09 0.31	0.00 0.96	−0.10 0.23	0.13 0.13	−0.02 0.84	0.08 0.34	0.48 <0.0001	0.51 <0.0001	0.47 <0.0001	0.48 <0.0001	0.50 <0.0001	0.48 <0.0001
Waist circumference	−0.60 <0.0001	−0.60 <0.0001	−0.59 <0.0001	−0.67 <0.0001	−0.66 <0.0001	0.69 <0.0001	0.69 <0.0001	0.78 <0.0001	0.76 <0.0001	−0.04 0.62	0.08 0.38	−0.19 0.03	0.17 0.05	−0.02 0.78	0.09 0.29	0.76 <0.0001	0.77 <0.0001	0.76 <0.0001	0.77 <0.0001	0.82 <0.0001	0.79 <0.0001
Hip circumference	−0.55 <0.0001	−0.51 <0.0001	−0.50 <0.0001	−0.58 <0.0001	−0.56 <0.0001	0.59 <0.0001	0.62 <0.0001	0.71 <0.0001	0.70 <0.0001	−0.07 0.40	0.06 0.47	−0.14 0.10	0.18 0.04	0.00 0.99	0.10 0.23	0.66 <0.0001	0.70 <0.0001	0.67 <0.0001	0.68 <0.0001	0.74 <0.0001	0.72 <0.0001

Triglycerides	−0.13 0.13	−0.15 0.08	−0.18 0.04	−0.24 0.01	−0.19 0.02	0.16 0.05	0.21 0.02	0.20 0.02	0.19 0.03	0.07 0.44	0.13 0.14	0.04 0.62	0.11 0.21	0.04 0.66	0.10 0.23	0.15 0.07	0.19 0.02	0.18 0.04	0.21 0.01	0.20 0.02	0.17 0.04
Cholesterol	−0.06 0.49	−0.06 0.46	−0.06 0.47	−0.08 0.36	−0.11 0.19	0.12 0.15	0.14 0.11	0.11 0.21	0.10 0.24	0.07 0.43	0.10 0.24	0.08 0.35	0.13 0.14	0.08 0.37	0.05 0.54	0.11 0.21	0.12 0.16	0.09 0.31	0.12 0.16	0.10 0.27	0.10 0.27
HDL	0.36 <0.0001	0.41 <0.0001	0.40 <0.0001	0.43 <0.0001	0.39 <0.0001	−0.28 0.001	−0.29 0.001	−0.33 <0.0001	−0.32 0.0001	0.11 0.20	0.02 0.81	0.20 0.02	0.01 0.95	0.11 0.19	0.02 0.81	−0.34 <0.0001	−0.35 <0.0001	−0.39 <0.0001	−0.36 <0.0001	−0.37 <0.0001	−0.35 <0.0001
Alkaline phosphatase	−0.22 0.01	−0.29 0.001	−0.26 0.002	−0.34 <0.0001	−0.35 <0.0001	0.37 <0.0001	0.42 <0.0001	0.46 <0.0001	0.44 <0.0001	0.10 0.25	0.18 0.03	−0.05 0.57	0.18 0.03	0.09 0.29	0.11 0.21	0.36 <0.0001	0.41 <0.0001	0.43 <0.0001	0.43 <0.0001	0.45 <0.0001	0.44 <0.0001
Alanine aminotransferase	−0.09 0.28	−0.11 0.19	−0.08 0.39	−0.11 0.19	−0.11 0.19	0.15 0.08	0.13 0.14	0.13 0.13	0.13 0.14	0.05 0.57	0.06 0.51	−0.02 0.86	0.06 0.51	0.05 0.58	0.06 0.51	0.14 0.09	0.13 0.14	0.13 0.13	0.13 0.12	0.13 0.15	0.12 0.16
Aspartate aminotransferase	0.12 0.17	0.09 0.31	0.14 0.11	0.08 0.33	0.10 0.25	−0.04 0.68	0.01 0.92	−0.04 0.68	−0.04 0.66	0.12 0.18	0.15 0.09	0.14 0.11	0.14 0.11	0.14 0.11	0.12 0.15	−0.08 0.35	−0.05 0.57	−0.07 0.42	−0.05 0.57	−0.07 0.43	−0.07 0.42

Sex hormone binding globulin	0.28 0.0010	0.33 <0.0001	0.32 0.0001	0.35 <0.0001	0.36 <0.0001	−0.41 <0.0001	−0.40 <0.0001	−0.43 <0.0001	−0.43 <0.0001	−0.10 0.27	−0.15 0.07	0.02 0.83	−0.16 0.06	−0.07 0.42	−0.17 0.05	−0.41 <0.0001	−0.40 <0.0001	−0.39 <0.0001	−0.42 <0.0001	−0.43 <0.0001	−0.42 <0.0001
C-reactive protein	−0.22 0.01	−0.31 0.0002	−0.23 0.01	−0.36 <0.0001	−0.35 <0.0001	0.50 <0.0001	0.56 <0.0001	0.60 <0.0001	0.59 <0.0001	0.20 0.02	0.30 0.0003	0.03 0.70	0.35 <0.0001	0.21 0.02	0.26 0.0019	0.46 <0.0001	0.53 <0.0001	0.52 <0.0001	0.53 <0.0001	0.57 <0.0001	0.57 <0.0001
Insulin	−0.20 0.02	−0.26 0.003	−0.18 0.04	−0.28 0.001	−0.28 0.001	0.39 <0.0001	0.43 <0.0001	0.46 <0.0001	0.45 <0.0001	0.15 0.08	0.23 0.01	0.04 0.68	0.26 0.00	0.15 0.09	0.22 0.01	0.37 <0.0001	0.41 <0.0001	0.39 <0.0001	0.40 <0.0001	0.44 <0.0001	0.43 <0.0001
IGF-I	0.15 0.09	0.21 0.02	0.21 0.01	0.22 0.01	0.28 0.001	−0.22 0.01	−0.18 0.03	−0.24 0.01	−0.24 0.004	−0.05 0.57	−0.06 0.51	0.06 0.52	−0.05 0.56	0.00 0.97	0.02 0.84	−0.22 0.01	−0.19 0.02	−0.21 0.01	−0.21 0.01	−0.24 0.004	−0.26 0.002
IGF-II	−0.12 0.17	−0.11 0.19	−0.16 0.07	−0.20 0.02	−0.18 0.03	0.24 0.004	0.25 0.004	0.26 0.003	0.25 0.004	0.07 0.40	0.10 0.26	0.02 0.84	0.09 0.31	0.03 0.72	0.02 0.83	0.23 0.01	0.25 0.003	0.23 0.01	0.27 0.00	0.26 0.002	0.26 0.002
17*β*-Estradiol	0.06 0.51	0.19 0.03	0.12 0.16	0.13 0.14	0.12 0.16	−0.08 0.33	−0.04 0.67	−0.09 0.28	−0.10 0.23	0.05 0.55	0.08 0.32	0.23 0.01	0.12 0.17	0.16 0.07	0.14 0.11	−0.11 0.20	−0.08 0.37	−0.17 0.05	−0.09 0.28	−0.13 0.13	−0.14 0.10
Progesterone	0.12 0.15	0.14 0.10	0.15 0.09	0.11 0.20	0.16 0.07	−0.12 0.15	−0.06 0.45	−0.09 0.28	−0.09 0.31	0.03 0.75	0.06 0.46	0.13 0.13	0.07 0.41	0.08 0.38	0.10 0.25	−0.14 0.09	−0.10 0.24	−0.14 0.11	−0.11 0.19	−0.11 0.18	−0.12 0.17

**Table 4:** Multivariate analysis model estimates for percent breast density (%-G), fibroglandular tissue volume (GV), fat tissue volume (FV), and total breast volume (TV) measured by five different methods (*n* = 137).

Explanatory variable	Standardized *β*-estimates (SE)			
	%-G	GV	FV	TV
BMI	−0.59 (0.09)∗∗∗	−0.10 (0.11)	0.65 (0.07)∗∗∗	0.52 (0.08)∗∗∗
Age	−0.09 (0.07)	−0.13 (0.08)	−0.09 (0.05)	−0.12 (0.06)∗
Age at menarche	0.13 (0.07)	0.13 (0.08)	−0.05 (0.05)	0.01 (0.06)
Pregnancy, completed				
Zero	Reference			
One	0.22 (0.26)	−0.29 (0.32)	−0.28 (0.21)	−0.34 (0.23)
Two	−0.52 (0.21)∗	−0.81 (0.27)∗∗	−0.08 (0.17)	−0.34 (0.19)
Three and more	−0.52 (0.22)∗	−0.99 (0.28)∗∗∗	−0.18 (0.18)	−0.49 (0.20)∗∗
Alkaline phosphatase (ALP)	−0.06 (0.08)	0.06 (0.10)	0.12 (0.06)∗	0.12 (0.07)
Alanine aminotransferase (ALT)	−0.22 (0.10)∗	−0.16 (0.12)	0.07 (0.08)	0.01 (0.09)
Aspartate aminotransferase (AST)	0.32 (0.10)∗∗∗	0.24 (0.12)∗	−0.15 (0.08)	−0.04 (0.09)
Insulin-like growth factor I (IGF-I)	−0.03 (0.07)	−0.10 (0.09)	−0.04 (0.06)	−0.07 (0.06)
Insulin-like growth factor II (IGF-II)	−0.10 (0.07)	0.03 (0.09)	0.16 (0.06)∗∗	0.15 (0.06)∗
Sex hormone binding globulin (SHBG)	0.07 (0.07)	−0.09 (0.09)	−0.05 (0.06)	−0.07 (0.06)
C-reactive protein (CRP)	0.13 (0.09)	0.23 (0.11)∗	0.05 (0.07)	0.13 (0.08)
17*β*-Estradiol	−0.09 (0.07)	0.01 (0.09)	0.02 (0.06)	0.02 (0.06)
Progesterone	0.13 (0.07)∗	0.20 (0.08)∗	−0.02 (0.05)	0.05 (0.06)
Measurement methods^#^				
Histogram segmentation method (HSM)	Reference			
Full field digital mammography (FFDM)	−0.02 (0.27)	−0.12 (0.33)	−0.10 (0.21)	0 (0.23)
Mathematical algorithm (MATH)	0.10 (0.27)	0.01 (0.33)	−0.01 (0.21)	0 (0.23)
3D gradient-echo MRI (3DGRE)	−0.04 (0.27)	−0.13 (0.34)	−0.01 (0.21)	−0.10 (0.24)
Short tau inversion recovery MRI (STIR)	−0.10 (0.27)	−0.14 (0.33)	−0.003 (0.21)	−0.09 (0.23)
Race and ethnicity				
Non-Hispanic White	Reference			
Hispanic	0.30 (0.16)	0.32 (0.20)	−0.11 (0.13)	0.02 (0.14)
African-American	0.40 (0.20)∗	0.34 (0.25)	−0.07 (0.16)	0.06 (0.18)
Model *R* ^2^	0.54	0.29	0.71	0.65

***P < 0.001, **P < 0.01, and *P < 0.05 for predictor strength within a regression model.

^#^All *P* values >0.05 for interaction terms between predictor variables and measurement methods (results not shown).

**Table 5:** Multivariate analysis model estimates for percent breast density (%-G), fibroglandular tissue volume (GV), fat tissue volume (FV), and total breast volume (TV) measured by five different methods (*n* = 320).

Explanatory variable	Standardized *β*-estimates (SE)			
	%-G	GV	FV	TV
BMI	−0.62 (0.02)∗∗∗	0.03 (0.03)	0.79 (0.02)∗∗∗	0.71 (0.02)∗∗∗
Age	−0.03 (0.2)	−0.01 (0.02)	−0.02 (0.02)	−0.02 (0.02)
Age at menarche	0.03 (0.2)	0.03 (0.02)	0.01 (0.02)	0.02 (0.02)
Pregnancy, completed				
Zero	Reference			
One	−0.08 (0.08)	−0.002 (0.1)	0.18 (0.07)∗	0.16 (0.07)∗
Two	−0.24 (0.07)∗∗	−0.37 (0.08)∗∗∗	0.005 (0.06)	−0.14 (0.06)∗
Three and more	−0.41 (0.07)∗∗∗	−0.69 (0.08)∗∗∗	−0.12 (0.06)∗	−0.35 (0.06)∗∗∗
Measurement method^a,b^				
HSM	Reference			
GE	−0.003 (0.06)	−0.01 (0.07)	0.002 (0.05)	0 (0.05)
MATH	0.01 (0.06)	0.01 (0.07)	0.001 (0.05)	0 (0.05)
3DGRE	0.002 (0.06)	−0.003 (0.07)	0.003 (0.05)	0 (0.06)
STIR	0.03 (0.06)	0.01 (0.08)	−0.02 (0.05)	−0.02 (0.06)
Race and ethnicity				
Non-Hispanic White race	Reference			
Hispanic race	0.16 (0.05)∗∗	0.33 (0.06)∗∗∗	−0.12 (0.04)∗∗	0.01 (0.04)
African-American race	0.47 (0.06)∗∗∗	0.39 (0.08)∗∗∗	−0.22 (0.05)∗∗∗	−0.05 (0.06)
Model *R* ^2^	**0.38**	**0.09**	**0.58**	**0.51**

^a^HSM, histogram segmentation method; FFDM, full field digital mammography unit; MATH, mathematical algorithm; 3DGRE, 3D gradient echo; STIR, short tau inversion recovery.

^b^Interaction terms between predictors and measurement methods were all not significant (results not shown).

***P < 0.001, **P < 0.01, and *P < 0.05.

## Abbreviations Used

BD: Breast density
HSM: Histogram segmentation method
MATH: Mathematical algorithm
FFDM: Full field digital mammography
MRI: Magnetic resonance imaging
%-G: %-Glandular tissue or %-breast density
GV: Glandular/fibroglandular breast volume
FV: Fat/adipose breast volume
TV: Total breast volume
3DGRE: a 3D gradient-echo MRI pulse sequence
STIR: A fat suppressing short tau inversion recovery MRI pulse sequence
TE: Echo time
TR: Repetition time
TI: Inversion time
FOV: Field of view
NEX: Number of excitations
BMI: Body mass index
ALT: Alanine aminotransferase
AST: Aspartate aminotransferase
ALP: Alkaline phosphatase
SHBG: Sex hormone binding globulin
CRP: C-reactive protein
IGF-I: Insulin-like growth factor I
IGF-II: Insulin-like growth factor-II.
